# Extended Reality (XR) in Pediatric Acute and Chronic Pain: Systematic Review and Evidence Gap Map

**DOI:** 10.2196/63854

**Published:** 2025-04-07

**Authors:** Courtney W Hess, Brittany N Rosenbloom, Giulia Mesaroli, Cristal Lopez, Nhat Ngo, Estreya Cohen, Carley Ouellette, Jeffrey I Gold, Deirdre Logan, Laura E Simons, Jennifer N Stinson

**Affiliations:** 1 Department of Anesthesiology, Perioperative, & Pain Medicine Stanford University School of Medicine Palo Alto, CA United States; 2 Toronto Academic Pain Medicine Institute Women's College Hospital Toronto, ON Canada; 3 Department of Anesthesia and Pain Medicine University of Toronto Toronto, ON Canada; 4 Department of Rehabilitation Services Hospital for Sick Children Toronto, ON Canada; 5 Department of Physical Therapy University of Toronto Toronto, ON Canada; 6 Research Institute Hospital for Sick Children Toronto, ON Canada; 7 The Saban Research Institute Children's Hospital Los Angeles Los Angeles, CA United States; 8 Department of Anesthesiology Critical Care Medicine Children's Hospital Los Angeles Los Angeles, CA United States; 9 Department of Psychology York University Toronto, ON Canada; 10 School of Nursing McMaster University Hamilton, ON Canada; 11 Department of Anesthesiology, Pediatrics, and Psychiatry and the Behavioral Sciences Keck School of Medicine University of Southern California Los Angeles, CA United States; 12 Department of Anesthesiology, Critical Care and Pain Medicine Boston Children's Hospital Boston, MA United States; 13 Department of Anesthesiology, Critical Care and Pain Medicine Harvard Medical School Cambridge, MA United States; 14 Lawrence S. Bloomberg Faculty of Nursing University of Toronto Toronto, ON Canada

**Keywords:** virtual reality, augmented reality, extended reality, acute pain, chronic pain, pediatrics, adolescents, safety, feasibility, effectiveness, evidence gap map, child, children, VR, XR, biobehavioral, intervention, systematic review

## Abstract

**Background:**

The use of extended reality (XR), including virtual reality (VR) and augmented reality (AR), for treating pain has accelerated in the last 10 years. XR is an attractive biobehavioral intervention that may support management of pain or pain-related disability. Reviews of the literature pertaining to adults report promising results, particularly for acute procedural pain.

**Objective:**

This study aimed to (1) summarize the available evidence with respect to feasibility, safety, and effectiveness (pain intensity) of XR for pediatric acute and chronic pain; (2) summarize assessment tools used to measure study outcomes; and (3) identify gaps in evidence to guide future research efforts.

**Methods:**

This study is a systematic review of the literature. Multiple databases (CINAHL, Cochrane Central, Embase, MEDLINE, PsycINFO) were searched from inception until March 2023. Titles, abstracts, and full-text articles were reviewed by 2 team members to determine eligibility. Articles were included if the (1) participants were aged 0 to 18 years; (2) study intervention was VR or AR; (3) study outcomes included safety, feasibility, acceptability, or effectiveness on the outcome of pain; and (4) study design was observational or interventional. Data were collected on bibliographic information; study characteristics; XR characteristics; outcome domains; outcome measures; and study findings pertaining to safety, feasibility, and effectiveness.

**Results:**

We included 90 articles in the review. All included studies used VR, and 93% (84/90) studied VR in the context of acute pain. Of the 90 studies, 74 studies were randomized trials, and 15 studies were observational. Safety was assessed in 23 studies of acute pain, with 13 studies reporting no adverse events and 10 studies reporting events of low concern. Feasibility was assessed in 27 studies. Of the 84 studies of acute pain, 62% (52/84) reported a positive effect on pain intensity, 21% (18/84) reported no effect, and 13% (11/84) reported mixed effects. All 6 studies of chronic pain reported a positive effect on pain intensity. An evidence gap map was used to illuminate gaps in specific research areas stratified by subtypes of pain. Risk of bias assessment revealed 67 studies had a moderate risk of bias, 17 studies had a high risk, and 5 studies were deemed to be low risk.

**Conclusions:**

The current body of literature around XR for pediatric pain is focused on acute pain with promising results of safety and effectiveness on pain intensity. The literature pertaining to chronic pain lags behind, limiting our ability to draw conclusions. The risk of bias in studies is problematic in this field, with the inherent challenge of blinding participants and researchers to the intervention. Future research should aim to measure effectiveness beyond pain intensity with a consistent approach to measuring key outcome domains and measures. Current efforts are underway to establish expert consensus on best research practices in this field.

**Trial Registration:**

Prospero CRD42022307153; https://www.crd.york.ac.uk/PROSPERO/view/CRD42022307153

## Introduction

Acute and chronic pain are common among children [1-3] and can result in negative short and long-term health and mental health consequences. Within pediatric populations, acute pain can negatively impact treatment adherence such as vaccination schedules [4] and routine port access [5] and can worsen outcomes in the context of surgery and rehabilitation [6,7]. Additionally, acute pain can transition into chronic pain if not adequately assessed, managed, and treated [8]. If not addressed in childhood, these pain and other health-related problems often continue into adulthood and therein increase risk for functional disability and mental health challenges including depression, suicidality, and substance use, with particular concern for opioid use or misuse [9-12].

Technologies have been emerging to support the management of pain for both acute and chronic pain, including the integration of extended reality (XR) into clinical care. XR is most often inclusive of virtual reality (VR) and augmented reality (AR), which aim to immerse a person or patient in a simulated environment that they perceive to be real [13]. In the context of VR, youth are transported to a new or alternative environment, while AR alters or enhances the environment in which the youth is already existing [14].

The integration of XR into health care has been a rapidly growing area of clinical research and treatment, with promise surrounding the management of pain across a variety of acute and chronic conditions for youth and adults [15-17]. Much research has been conducted in the acute pain management context and specifically surrounding wound care, venipuncture (“needle pokes”), and minor procedures (eg, dental fluoride therapy, orthopedic pin removal). Among adults, XR has also been used commonly to manage chronic pain such as low back pain or fibromyalgia [18,19]. Together, results to date indicate that XR holds great promise for supporting biopsychosocial treatment for pain management for youth and adults; however, consistency in measurement as well as defined outcomes for assessing the effectiveness of XR research are missing, thereby limiting the ability to coordinate research efforts and examine effectiveness on a large scale.

One reason for the variability and the lack of consensus in technology and outcomes in XR research is related to the rapid adoption and evolution of technology (ie, hardware and software). Since the initial studies of XR for pain began in the 1980s, the rate at which XR is being evaluated and integrated into clinical care for pain management has sharply increased, with over 250 PubMed-indexed studies published since 2020 alone compared with the total 78 studies published in the 20 years preceding. Identifying a core outcome set for XR trials in pain is needed given the rapid adoption and evolution of this technology into clinical research and the potential for XR to provide breakthroughs in the treatment and management of acute and chronic pain. We undertook this task and, as a part of this effort, conducted a systematic review to summarize the current state of the literature with a specific aim toward understanding how researchers are currently assessing XR effectiveness.

Therefore, the purpose of this systematic review was to describe the feasibility, safety, and effectiveness of XR for pediatric acute and chronic pain. Moreover, this review collated current research to establish an evidence and gap map for guiding future research efforts. Finally, given the variability in XR study treatment outcomes, this review also assessed the measurement tools that have been used to measure outcomes in XR research. Together, these findings provide an updated description of the state of the field and highlight a path forward for improving the application, feasibility, and effectiveness of XR research in the context of pediatric acute and chronic pain.

## Methods

### Search Strategy

The systematic review protocol was registered on PROSPERO (#CRD42022307153). The literature search strategy was developed in consultation with a medical librarian who executed the searches. Examples of search terms (including MeSH terms) included “virtual reality,” “pain,” “treatment outcome,” “safety,” and “feasibility studies.” A randomized controlled trial (RCT) hedge or study-type filter was not applied as it was considered too limiting for the overall search. Major electronic databases (CINAHL, Cochrane Central, Embase, MEDLINE, PsycINFO) were searched from database inception to March 2023. The full search strategy is included in Multimedia Appendix 1.

### Selection Criteria

The inclusion criteria for the current review included (1) participants aged 0 to 18 years; (2) study intervention of XR (VR or AR); (3) study outcomes of safety, feasibility, acceptability, or effectiveness on the outcome of pain; and (4) observational or interventional study design. For this review, XR was defined as “technology that blurs the lines between the physical and virtual worlds, creating a sense of immersion and enhancing the realism of virtual experiences” [20-22]. Additionally, the age criterion was not applied until screening to allow for a comprehensive search of the literature.

Studies were excluded from the review if they were reviews, opinion papers, case studies with ≤4 participants, or conference proceedings. Studies in which the participant sample was exclusively adults (>18 years old) were excluded as well as studies in which the intervention was not deemed to align with the definition of XR (eg, intervention delivered on a computer screen or flat board display panel) or where there was insufficient description of the hardware to determine whether the intervention met the definition of XR. Although no uniform definition nor criterion exists for defining XR as immersive or not immersive, through group discussion and guided by existing definitions [23], we excluded technology where the user only existed in the real world (eg, looking at a 2D image on a computer). If a reviewer was uncertain about whether the XR trial was immersive, additional review of the study and group discussion were used to determine whether the paper was included in the review. Papers that were not available in the English language were also excluded from the review.

### Study Selection

Following completion of each of the systematic literature searches, 2 reviewers independently screened titles and abstracts for inclusion following established PICO (Population, Intervention, Comparison, Outcome) strategies. Disagreements between raters were resolved by a third reviewer. Following initial review of title and abstracts, a subsequent review of retained studies was completed, reviewing complete texts to evaluate for inclusion. Disagreements between raters were resolved by the same third reviewer. All screening was completed through Covidence [24], a web-based collaboration software platform that streamlines systematic review management and allows for blinded, independent review of included studies.

### Assessment of Study Quality

#### Assessment Tools

For studies that assessed the effectiveness of the XR intervention, a study risk of bias assessment was conducted. For RCTs, the Cochrane Risk of Bias Tool (RoB-2) was used, and for nonrandomized studies, the Methodological Index for Non-Randomized Studies (MINORS) was used. The risk of bias assessment was completed by 2 separate groups with one group conducting risk of bias for the RCTs and the other group conducting risk of bias for the nonrandomized trials. Given the distinct tools used for each group, it was deemed appropriate to allow for 2 separate coding groups.

#### Risk of Bias in RCTs

To assess the RCTs, the 2 reviewers used and adhered to the RoB-2 protocol [25]. RoB-2 serves as an algorithmic framework for considering the risk of bias in the outcomes of RCTs [26]. The tool asks reviewers to rate the bias in RCT protocols among 5 domains: (1) randomization process, (2) deviations from intended interventions, (3) missing outcome data, (4) measurement of the outcome, and (5) selection. Reviewers evaluated the studies independently then met to discuss agreement on rating. If the raters disagreed on a rating, a third rater resolved the disagreement. Each domain was assigned an algorithm result and an assessor’s judgement of Low, Some Concerns, or High. The overall bias followed the same 3 outcomes.

#### Risk of Bias in Nonrandomized Studies

To assess the nonrandomized studies (eg, observational, interventional without control), the reviewers used MINORS [27]. MINORS consists of 12 method-oriented items (eg, “A clearly stated aim”) to assess quality and risk of bias in nonrandomized studies. Each item is rated on a scale from 0 to 2, with 0 indicating that the item was not reported, 1 indicating that the item was reported but inadequate, and 2 indicating the item was reported and adequate. MINORS can be used for studies with or without a comparator group, with the first 8 items applying to all studies and the final 4 items specific to comparative studies. Total scores for noncomparative studies range from 0 to 16, and total scores for comparative studies range from 0 to 24, with higher scores indicating more methodological integrity. For this review, 2 raters independently rated each of the nonrandomized studies then met to compare. Each of the 12 items as well as the total scores were compared between raters. Any disagreements were resolved through discussion and, if needed, through consultation with a third rater.

### Data Extraction

A data extraction form was created and pilot tested to ensure relevant data were pulled based on the form directions. A team of research assistants was trained on the data extraction form, and each research assistant independently extracted data for their assigned studies. Data extracted included study characteristics (participants, design, setting, control group), intervention characteristics (hardware, software, intervention protocols), outcome domains (safety, feasibility, effectiveness), outcome measures used, and a summary of study findings.

### Data Coding (Evidence Gap Map)

All included studies were uploaded to EPPI Reviewer, software designed for systematic reviews and in support of developing evidence and gap maps. Using existing literature, authors CWH and GM developed operational definitions for each of the pain populations and target outcomes, which were reviewed by the larger author group and iterated until a final draft was created then used to guide coding of the studies. To code studies for the evidence and gap map, reviewers were assigned a series of studies in EPPI Reviewer and were asked to review the manuscript and code each article for the patient population (adult or pediatrics), pain population, and outcomes targeted by VR. Two training meetings were held with all reviewers to provide operational definitions, and they were asked to code a single article. A subsequent meeting was held to provide feedback and answer questions to increase consistency across coding. Once all articles were coded, any articles identified as unclear were reviewed again by a single author. Operational definitions are provided in Multimedia Appendix 2.

### Data Synthesis

Included studies were summarized, and fulsome details of all included studies are provided in Multimedia Appendix 3. Additionally, descriptive statistics were used to synthesize study characteristics and aims as well as intervention characteristics. To synthesize data pertaining to outcome domains, an evidence and gap map was created. To create the evidence and gap map, 7 reviewers independently coded each study according to the study population including age (pediatric, adult) and pain type (acute pain-venipuncture, acute pain-wound care, acute pain-procedural, acute pain-other, chronic pain-cancer related, chronic pain-postsurgical/trauma, chronic pain-headache/migraine, chronic pain-musculoskeletal, chronic pain-neuropathic, chronic pain-other), as well as the pain-related outcomes targeted by the XR intervention (user experiences, participation/engagement, cognitive, behavioral, physical functioning, pain intensity, quality of life, health care utilization, safety, feasibility). Each study was also coded according to its established risk of bias (low, moderate, high). Coding was conducted through EPPI Reviewer [28], a collaborative web-based research synthesis software that supports study classification, and EPPI Mapper, a web-based program that generates evidence and gap maps based on coding conducted in EPPI Reviewer. Finally, for each of the identified pain treatment targets, the measures used to assess those targets were extracted by 2 authors and summarized to indicate the number of unique measures reported for each treatment target, and the number of studies that used the measure.

## Results

### Article Identification

**Figure 1:**
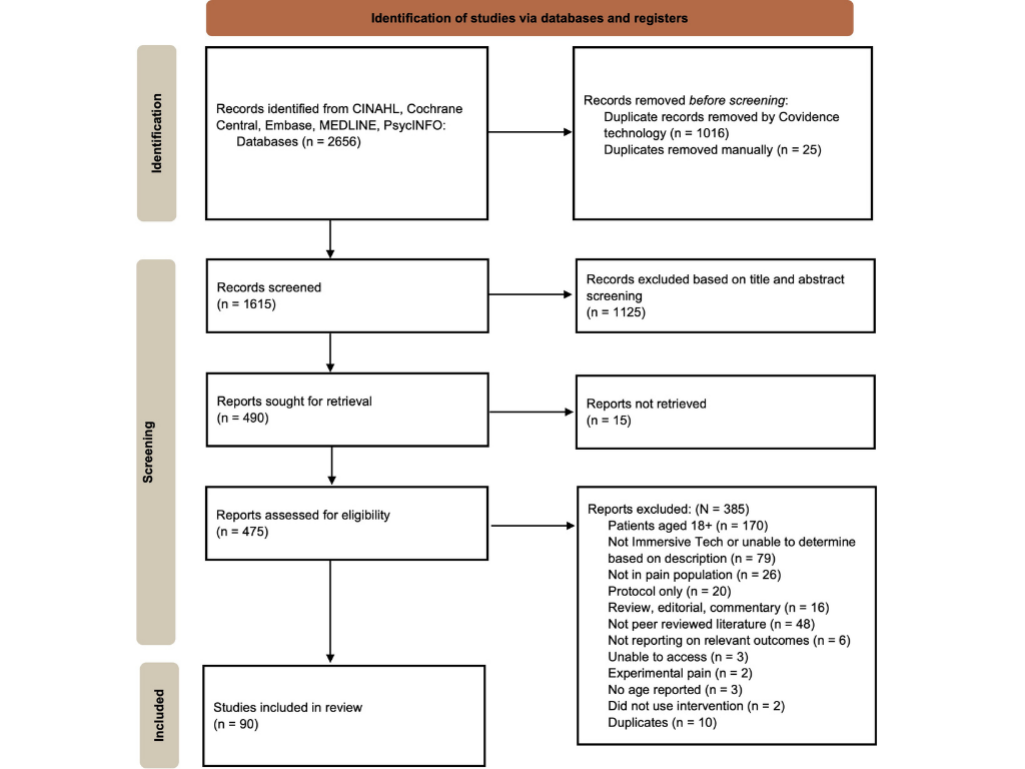
PRISMA (Preferred Reporting Items for Systematic Reviews and Meta-Analyses) flow diagram.

The search identified 2656 articles across the 5 identified databases. Of the articles identified, 1041 duplicates were identified and removed prior to abstract and title screening. We screened 1615 articles for inclusion based on abstract and title, and 1125 were removed based on relevancy to the review. Of the remaining 490 studies, 15 could not be located, so 475 full-text articles were screened for inclusion. Of those, 90 studies were included in the review. The most common reason for exclusion was that the study sample was exclusively adult (170 studies) followed by determination that the XR technology was not immersive (79 studies). See Figure 1 for additional information related to the screening process.

### Study Characteristics

**Figure 2:**
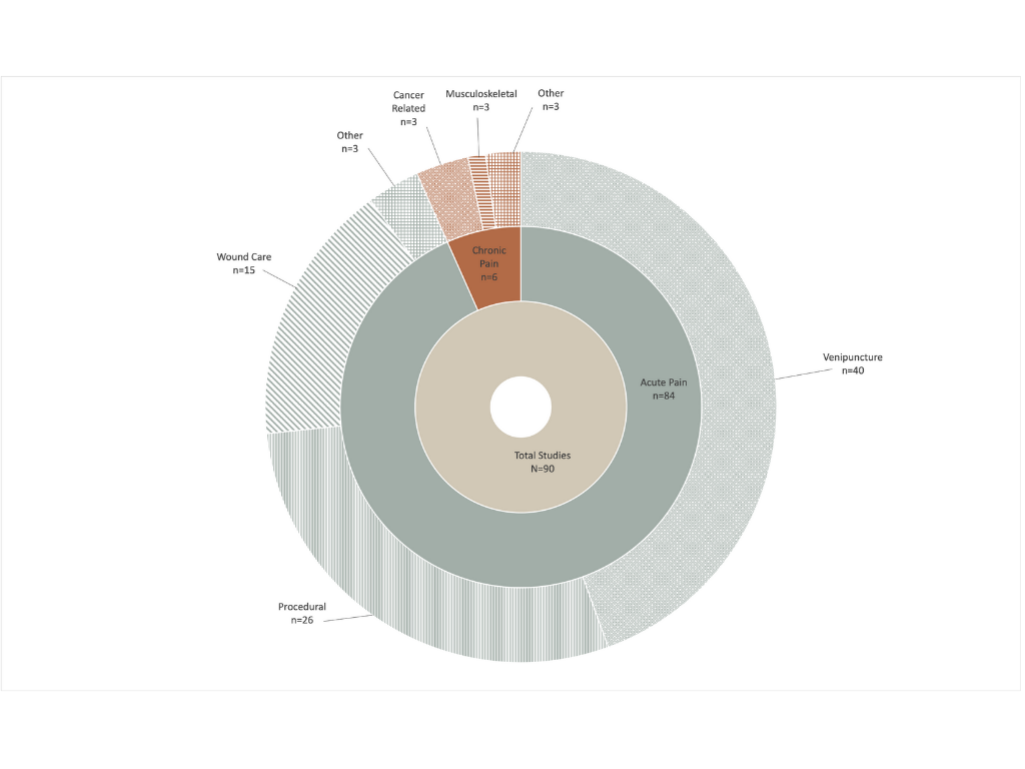
Pain types across all included studies.

Of the included articles, all 90 studies used VR technology, and no studies reported the use of AR technology. As such, when discussing XR for pediatric pain management, currently this is exclusively VR technology. Additionally, the majority of studies focused on assessment of VR in pediatric acute pain (84/90, 93%), specifically venipuncture (40/90, 48%) [29-68], burn dressing and wound care (15/90, 18%) [69-83], and minor procedures (26/90, 31%) [31,84-108], as well as other acute pain (3/90, 3%; ie, acute pain in emergency department, acute upper limb rehabilitation, vaso-occlusive crisis) [109-111]. A much smaller number of studies (n=6) examined the utility of VR in chronic pain populations including intensive pain rehabilitation (n=1) [112], chronic burn dressing (n=1) [113], chronic musculoskeletal pain (n=1) [114], chronic cancer-related pain (n=2) [115,116], and chronic abdominal pain (n=1) [117]. See Figure 2 for the summary of pain populations included across the studies. Within the 90 included studies, there were a total of 6596 participants enrolled, including 3615 enrolled in intervention arms and 2981 enrolled in control arms. Study sample sizes ranged from 5 to 254. Across studies, 3631 participants were male, while 2929 were female. The age of the participants ranged from 3 years to 18 years. Among participants enrolled in the intervention group, the mean age was 11.07 (SD 2.64) years, and in the control groups, the mean age was 10.38 (SD 2.37) years. Most studies (84/90, 93%) evaluated the use of XR in the context of pediatric acute pain management, while 7% (6/90) of the studies focused on XR in the context of pediatric chronic pain. Most studies took place in a hospital setting (63/90, 70%), and interventions were most often delivered by a researcher (36/90, 40%) or nurse (19/90, 21%). Regarding intervention design, an RCT was used 82% (74/90) of the time. See Table 1 for a full description of the study characteristics.

**Table 1:** Study characteristics (n=90).

Characteristic			Studies, n (%)
**Acute pain (n=84)**			
	Venipuncture	40 (45)	
	Procedural	26 (32)	
	Wound care	15 (18)	
	Other	3 (5)	
**Chronic pain (n=6)**			
	Cancer related	2 (33)	
	Musculoskeletal pain	1 (17)	
	Other	3 (50)	
**Study design**			
	Interventional RCT^a^	74 (82)	
	Interventional no control	13 (14)	
	Case series	1 (1)	
	Prospective observational	2 (2)	
**Study setting**			
	Hospital/burn center	69 (77)	
	Research	2 (2)	
	Dental	9 (10)	
	Home	1 (1)	
	Clinical	9 (10)	
**Sample size (n=** **6596)**			
	VR^b^ intervention	3615 (55)	
	Control	2981 (43)	
**Intervention delivered by**			
	Registered nurse	19 (21)	
	Physical therapist	2 (2)	
	Dentist	1 (1)	
	Other clinician	2 (2)	
	Researcher	36 (40)	
	Caregiver	1 (1)	
	Unknown	29 (32)	

^a^RCT: randomized controlled trial.

^b^VR: virtual reality.

### Study Quality Assessment

**Figure 3:**
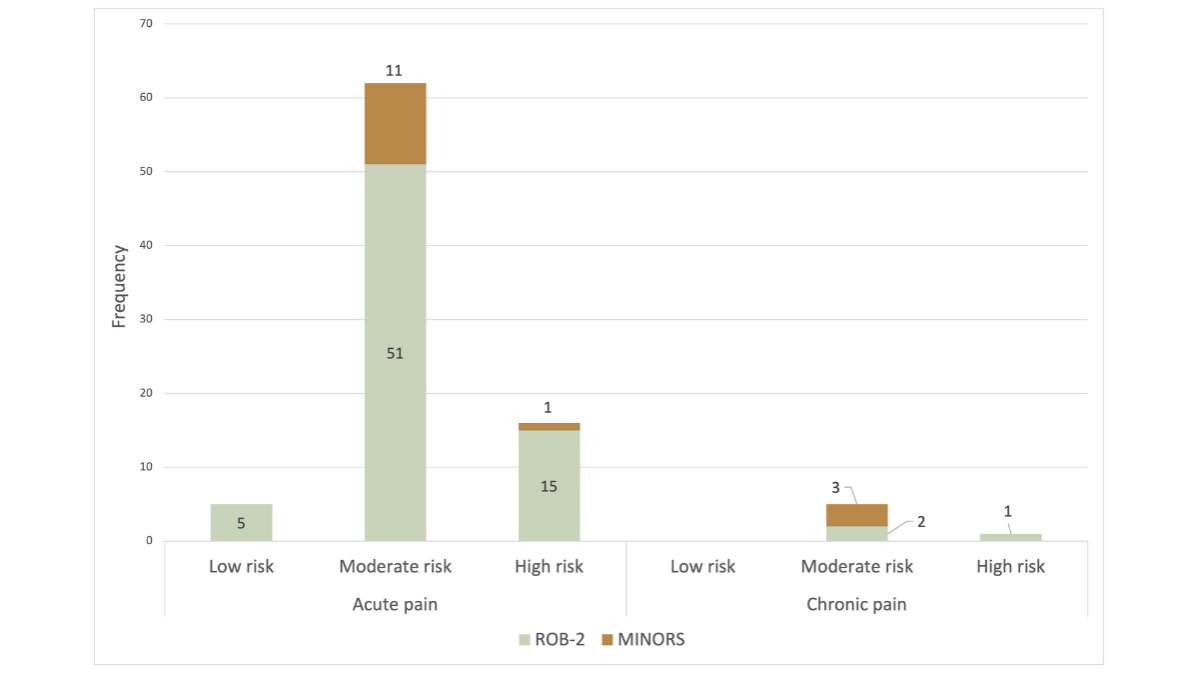
Risk of bias for included studies (N=89). MINORS: Methodological Index for Non-Randomized Studies; RoB-2: Cochrane Risk of Bias Tool.

Where possible, studies were examined for risk of bias based on study design. Of the 90 studies, 74 studies were evaluated using the RoB-2 assessment for randomized trials and 15 studies were evaluated using the MINORS assessment for nonrandomized interventional and observational studies. We could not assess 1 study due to the study design (ie, case series: Birnie et al [34]). Results of the risk of bias assessment revealed that most included studies (67/90, 74%) had a moderate risk of bias, while 17 studies (17/90, 19%) had a high risk of bias, and 5 studies (5/90, 6%) were deemed to have a low risk of bias. To see the summary of risk of bias ratings for each study, please see Figure 3. Although sources indicating risk of bias were variable across studies, most included studies were identified as moderate or high risk of bias due to their failure to blind study participants to the intervention and researchers to who was in the intervention arm (69 studies). Increased risk of bias was also commonly identified in the randomization procedures, although to a much lesser degree (19 studies).

### XR Feasibility

Across all included studies, feasibility was evaluated 43% (39/90) of the time. Within chronic pain studies, 50% (3/6) of the studies assessed the feasibility of VR. Although limited in scope, of the 3 studies that examined feasibility, results did indicate VR to be a positive experience that supported their management of pain. Within the acute pain studies (n=84), feasibility was assessed in 35 studies. When evaluated, feasibility of VR appears to be high according to patients, caregivers, and health care staff. Feasibility was assessed in several different ways including but not limited to recruitment and withdrawal rates, satisfaction surveys, patient-reported “fun,” and provider-reported disruption in clinical flow. Recruitment rates varied but indicated moderate (40/65, 62%) to high (66/71, 93%) levels of interest in VR use, and dropout rates were reported to be low. Health care professionals also indicated good feasibility. VR was perceived to not interfere with procedure time, decrease the perceived difficulty of procedures, and be easily implementable in the clinic setting, and health care staff expressed interest in repeated use. Patients and caregivers reported ease of use, high levels of satisfaction with VR, preference for VR over alternate distraction methods, and interest in repeated use in future procedures. As such, although more consistent assessment of VR feasibility is needed, the available data from the pediatric acute pain management setting suggest that VR is acceptable and feasible across stakeholders.

### XR Safety

Across included studies, safety, often measured through the presence of adverse events, was measured or reported 49% (44/90) of the time. Within the chronic pain studies, only 2 studies examined the safety of the VR intervention, so conclusions regarding the safety of VR for chronic pain populations cannot be made. Among the pediatric acute pain studies, 44 studies examined safety, limiting our ability to draw robust conclusions for safety. However, when safety or adverse events were reported, most studies (31/44, 70%) reported no adverse events or no differences between their control and VR intervention groups. When adverse events were reported, they were most often mild in nature and occurred in a minority of the participants. Rates of adverse events varied, occurring in <1% to 17% of participants, with intensity and severity of the concern low (eg, nausea rating of 4/100). Two studies reported that at least 1 participant withdrew from the intervention due to reported adverse events.

### XR Effectiveness

Several treatment targets emerged through the review as relevant to the effectiveness of VR for pain management. That is, targets of VR interventions for acute and chronic pain often went beyond pain intensity itself, as measures of effectiveness spanned several domains (eg, pain intensity, physical functioning, fear, anxiety). Given the wide variety of targeted outcomes, we summarized the findings across the identified target domains for acute and chronic pain. The variability in targets highlights several areas where additional research is needed to adequately examine the effectiveness of VR for pediatric pain.

**Figure 4:**
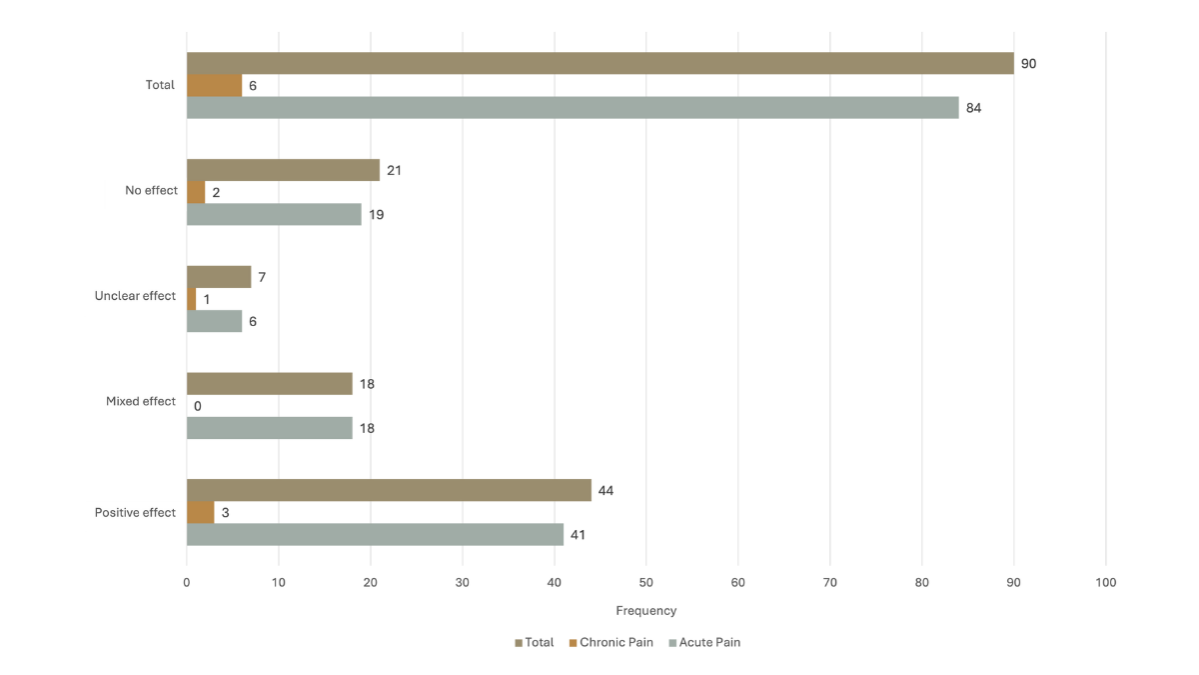
Effects of virtual reality on pain across pediatric acute and chronic pain.

Pain intensity was examined across all included studies (n=90), and overall, VR interventions demonstrated good effectiveness for reducing pain intensity (see Figure 4). Among the acute pain studies, 63% (53/84) demonstrated a positive effect (statistically significant reduction in pain) of VR on pain intensity, 21% (18/84) demonstrated no effect (nonsignificant reduction in pain or no change in pain), and 12% (10/84) demonstrated a mixed effect (different results across pain measures, pain reporter, or at different time points in the intervention) on pain intensity. Mixed effect findings were often due to a variable impact of VR on pain across a procedure (eg, positive impact on postprocedural pain but no effect on pain during the procedure) or variable impact of VR on pain across informants (eg, child reported improved pain, no improvement in pain reported by health care provider). No studies reported worse pain than the control or following the VR intervention. Among the chronic pain studies, 100% (6/6) of the studies demonstrated improved pain intensity in the context of the VR intervention.

### Evidence and Gap Map

**Figure 5:**
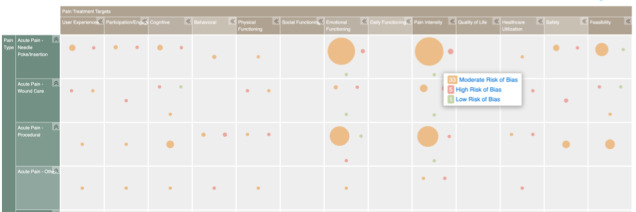
Snapshot of the evidence and gap map for virtual reality trials in pediatric acute and chronic pain [118]. The interactive evidence and gap map can be found in [119].

There was a wide range of outcome targets across studies. We created an evidence and gap map to collate current targets of VR in pain management as well as illuminate gaps (Figure 5). Guided by the conceptual model described by Trost et al [118], we categorized studies according to their target population as well as measured outcome targets. Additionally, the risk of bias results were integrated into the evidence and gap map. Therefore, the map illustrates, across the 90 included studies, the populations and pain targets with greater amounts of research evidence and those with missing or scant research evidence as well as the quality of evidence at various intersections (eg, pediatric venipuncture research targeting anxiety and pain intensity). Specifically, for XR research, the gap map illuminated significant gaps in research using XR to support pain management in pediatric chronic pain populations. Within the chronic pain populations, no research exists related to postsurgical or trauma-related pain, headaches, or neuropathic pain. Additionally, the risk of bias across all studies was moderate to high, prompting the need for more sophisticated research designs to reduce the risk of bias, particularly as it relates to blinding the research team and study participants. Within the acute pain setting, the most studied patient populations were children undergoing venipunctures and minor procedures. Primary targets of XR intervention for acute pain include pain intensity and emotional functioning. Across acute and chronic pain, measuring the effectiveness of XR on other important outcomes such as user experience, social functioning, and quality of life are important gaps that emerged from this review.

### Measures in XR Research

Across all study outcomes (feasibility, safety, effectiveness targets), there was tremendous variability in the outcome measures used. As such, we identified the number of distinct measures and the frequency of measures used across each outcome, which are summarized in Table 2. Full details of the measures used within each outcome domain can be found in Multimedia Appendix 4. Measures in XR research include validated, study-specific, researcher-developed and researcher-adapted tools. Additionally, similar measures are often used in distinct ways to measure different outcomes. For example, some researchers used heart rate as a measure of physical functioning, while others used heart rate as a measure of anxiety. The outcome target with the highest number of measures was psychological constructs, which contained 31 distinct measures across 67 studies. This is unsurprising as psychological construct is a broad outcome inclusive of multiple psychological and emotional states including but not limited to anxiety, mood, fear of pain, and pain catastrophizing. The outcome target with the second highest number of measures was feasibility, which contained 14 distinct measures across 28 studies, followed by user experience, which contained 13 distinct measures across 18 studies. The number of measures used across studies for each outcome domain is summarized in Table 2. This review highlighted that there is little agreement regarding how best to measure outcomes in XR research, an important area for future study, particularly given the high number of researcher-developed or researcher-adapted measures currently being used.

**Table 2:** The number of measures used within each outcome domain across pediatric acute and chronic pain studies.

Outcome domain	Measures, n
Pain intensity	21
Adverse events	8
User experience	13
Psychological constructs	31
Pain interference	8
Feasibility	14
Health care utilization	9
Quality of life	2
Physiological markers	10

## Discussion

### Principal Findings

Overall, in the context of pediatric acute pain, specifically venipuncture and minor procedural pain, good evidence exists to support continued use of XR, as it is effective for the management of pain and emotional functioning and demonstrates good feasibility and safety. Limited evidence exists, however, to guide the use of XR research in chronic pain populations. Although initial evidence does appear promising for the utility of XR to support pain management in several chronic pain populations (eg, cancer-related pain, abdominal pain, diffuse musculoskeletal pain), more robust examination is needed as is research that assesses the safety and feasibility of implementing XR into the flow of clinical care. Another area requiring more research in pediatric populations is the subacute period (ie, up to 3 months after injury, trauma, or surgery). Research has demonstrated this period to be a vulnerable time for the development of fear avoidance related to pain and movement, which increases the risk for the transition of acute pain to chronic pain [8]. XR interventions would likely be useful during this period as both a form of distraction and to support confrontation of feared movements.

This review indicates that most studies to date have been implemented within the context of a children’s hospital, which is unsurprising given the preponderance of research focused on acute pain management. However, there is limited research to guide the implementation of XR research outside the context of a hospital setting, which may be particularly important for chronic pain patients who may be receiving care across a variety of treatment settings (eg, home, outpatient clinic, physical therapy clinic). In the adult literature, XR has been successfully implemented into community care for chronic pain management [120,121]; however, this is unstudied in pediatric populations. The use of implementation science frameworks (eg, Consolidated Framework for Implementation Research) [122] to evaluate contextual and personnel differences as they relate to technology integration in settings outside of a hospital can support these efforts.

This review also demonstrated that research to date has been exclusively on the use of VR in pediatric pain management, and, as such, little is known about the potential for AR to support acute and chronic pain. Exploration of the utility of AR is particularly relevant in the context of chronic pain where exposure to painful movements may benefit from gradation such that a young person is first fully distracted by immersion into a VR world then gradually progressed toward exposure of their environment using an AR system to continue to support distraction while increasing exposure to their lived environment [123-125].

Another finding of this review is the significant variability in the way outcomes are being measured across XR studies. That is, measurement remains an area for needed consensus to support a better ability to compare effectiveness of XR across studies. Moreover, efforts are needed and indeed underway to better define both process (eg, immersion) [23] and outcome (eg, pain intensity) variables in XR trials, which will facilitate better reporting and thus improved ability to effectively compare XR across studies. Moreover, definitions of emotional functioning were also quite varied across studies, even when similar constructs were being targeted (ie, anxiety, fear, mood). Different definitions of anxiety and fear emerged across studies as well as how they were operationalized and assessed. For example, some studies assessed anxiety vis-à-vis heart rate variability and oxygen saturation, while other studies completed self-report assessments, proxy reports, and behavioral observations. Although all approaches to assessing anxiety may be valid, it limits the comparability of anxiety as an outcome across studies. Additionally, mood was examined to a much lesser degree in the reviewed studies and warrants increased attention, particularly in the context of chronic pain or acute-to-chronic pain transition. Another challenge with comparing XR effectiveness across studies is inconsistent reporting of study protocols. In this review, several studies were excluded because of an insufficient description of the XR technology. Creation of standards for reporting for XR studies, including how to describe the technology used (eg, hardware and software) and intervention protocol (eg, number of sessions, duration of intervention), is needed to increase the ability to replicate studies and compare outcomes across studies, thereby improving our ability to understand which XR approaches are most effective, when they are most effective, and for whom they are most effective.

There are also potential outcomes or targets of XR research that warrant attention, namely social and academic functioning alongside quality of life. Given the growing feasibility of real-time social interactions in the context of XR alongside established challenges for youth with pain to engage socially with peers [126], examination of the potential for XR research to improve or support social functioning is an important area for future work. Building from existing literature that has demonstrated the effectiveness of virtual peer-to-peer pain groups [127-130], development of virtual pain groups that can target multiple domains of functioning (eg, physical functioning, social functioning) is an untapped and potentially high-yield target for XR research. Similarly, academic functioning is commonly cited as a challenge for youth with chronic pain [131,132], and although research is emerging in this domain [133], more is needed to evaluate the potential for XR research to support academic engagement and facilitate a return to school learning for youth with pain. Finally, quality of life was minimally examined in the included studies (n=2). This is likely due to the overwhelming number of studies focused on acute pain where quality of life measures may be less relevant; however, development of quality of care measures or consideration for implementation of quality of care measures that are relevant to the acute pain context may be important to truly capture the impact of XR on patients. Moreover, as additional research is conducted in the chronic pain population, where pain and anxiety may become less of an emphasis, quality of life measures will be important markers of improvement.

Finally, decreasing the risk of bias in XR studies also emerged as an important priority for future research. That is, most studies had a moderate to high risk of bias according to the RoB-2 assessment, prompting the need for more sophisticated research designs. Specific areas where innovation is needed are addressing participant blinding to the intervention and the development of prespecified analysis plans. Given the nature of XR interventions, innovation for blinding participants to their assigned group, such as the development and use of sham XR software, is needed to decrease the risk of bias. Prepublished analysis plans and transparency surrounding blinding of the analyses are also needed.

### Limitations

This systematic review has some limitations that are important to note. First, the search for this systematic review was completed in March 2023 and likely omits several manuscripts published since then. Given the rapidity of XR research in pediatrics, it was not feasible for this research group to keep pace with the rate of publication. Moreover, this review was conducted with the aim of supporting the consensus conference surrounding outcomes for pediatric XR research. Future updates to this review are needed to support ongoing identification of gaps in research and inform clinical practice. Additionally, although a strength of this review is the breadth of studies included, future research could be conducted to allow for more focused assessment of efficacy, particularly as it relates to specific patient populations and XR configurations. To this end, future research should consider the interaction of XR interventions and child development to assess whether developmental stage or chronological age impact XR feasibility, safety, or effectiveness and guide precision medicine to match the XR intervention or configuration to best meet the needs of youth across diverse developmental stages. This review assessed the risk of bias for all included studies; however, this is not synonymous with quality and should be interpreted with caution. That is, studies may have been of high quality yet received high risk of bias scores due to their lack of blinding, a common challenge faced by XR researchers. Finally, studies were limited to the English language and excluded gray literature and conference papers, which may limit the inclusion of important research published in languages other than English and could reinforce publication bias such that studies in which XR was unhelpful or not feasible are not well represented.

### Conclusion

This systematic review provides an important update regarding the state of XR research for pediatric acute and chronic pain. Review of XR feasibility, safety, and effectiveness solidified the significant potential of XR for pain management across a variety of pain presentations and for a range of diverse outcomes. Moreover, the developed evidence and gap map illuminated important gaps in the current research base that warrant attention. Finally, limitations identified in the research studies reviewed also highlight the need for innovative research designs, establishment of measurement consensus, and improved reporting standards for XR studies to more effectively establish best practices in XR intervention research and improve clinical translation of the evidence base.

## Appendix

Multimedia Appendix 1 Librarian search strategy.

Multimedia Appendix 2 Operational definitions of pain populations and outcome targets to guide evidence and gap map coding.

Multimedia Appendix 3 Fulsome details of 90 included VR intervention studies for pediatric acute and chronic pain.

Multimedia Appendix 4 Measures used across outcome domains in VR trials for pediatric acute and chronic pain.

Multimedia Appendix 5 PRISMA (Preferred Reporting Items for Systematic Reviews and Meta-Analyses) checklist.

## Abbreviations

AR: augmented reality
MINORS: Methodological Index for Non-Randomized Studies
PICO: Population, Intervention, Comparison, Outcome
RCT: randomized controlled trial
RoB-2: Cochrane Risk of Bias Tool
VR: virtual reality
XR: extended reality

## Data Availability

All data from the systematic review are available upon request from primary or senior authors.
