# Supplementary material for: Extended Reality (XR) in Pediatric Acute and Chronic Pain: Systematic Review and Evidence Gap Map
Source: JMIR Pediatr Parent. 2025 Apr 7;8:e63854. doi: 10.2196/63854 (PMC12012403; doi:10.2196/63854)
Supplement: Multimedia Appendix 1 [file pediatrics_v8i1e63854_app1.docx]

| **Database [Platform]** *Searches run February 27, 2023* | **Results** |
| --- | --- |
| MEDLINE(R) and Epub Ahead of Print, In-Process & Other Non-Indexed Citations and Daily [OVID] 1946 to February 24, 2023 | 351 |
| Embase Classic+Embase [OVID] 1947 to 2023 Week 8 | 514 |
| EBM Reviews - Cochrane Central Register of Controlled Trials [OVID] January 2023 | 135 |
| APA PsycInfo [OVID] <1967 to February Week 2 2023> | 21 |
| CINAHL Plus [EBSCO] February 27, 2023 | 132 |
| **TOTAL** | **1153** |

**MEDLINE(R) and Epub Ahead of Print, In-Process, In-Data-Review & Other Non-Indexed Citations and Daily 1946 to February 24, 2023
Search Strategy:**

| **#** | **Searches** | **Results** |
| --- | --- | --- |
| 1 | virtual reality/ | 5176 |
| 2 | Virtual Reality Exposure Therapy/ | 858 |
| 3 | (virtual realit* or VR or augmented realit* or immersive technolog*).tw,kf. | 24853 |
| 4 | or/1-3 | 25746 |
| 5 | exp Pain/ | 451488 |
| 6 | exp Complex Regional Pain Syndromes/ | 5921 |
| 7 | Headache/ | 30991 |
| 8 | exp Headache Disorders/ | 39537 |
| 9 | (pain* or neuralgia* or complex regional pain syndrome? or CRPS or headache? or head ache?).tw,kf. | 917867 |
| 10 | or/5-9 | 1069622 |
| 11 | 4 and 10 | 1497 |
| 12 | treatment outcome/ | 1136636 |
| 13 | Safety/ | 41873 |
| 14 | Risk Assessment/ | 304103 |
| 15 | Equipment Safety/ | 10446 |
| 16 | Feasibility Studies/ | 82021 |
| 17 | Pain Management/ | 40317 |
| 18 | (effective* or feasible or feasibility or outcome? or safe or safe* or adverse event? or adverse effect? or harm* or risk? or undesirable effect? or undesirable event? or unexpected effect? or unexpected event?).tw,kf. | 7313052 |
| 19 | ((pain* or headache? or head ache?) adj3 (decreas* or manag* or reduc*)).tw,kf. | 106956 |
| 20 | or/12-19 | 7871339 |
| 21 | 4 and 10 and 20 | 1175 |
| 22 | limit 21 to english language | 1152 |
| 23 | limit 22 to dt=20220111-20231231 | 295 |
| 24 | limit 22 to ez=20220111-20231231 | 292 |
| 25 | limit 22 to ed=20220111-20231231 | 218 |
| 26 | or/23-25 | 354 |
| 27 | remove duplicates from 26 | 351 |

**Embase Classic+Embase**1947 to 2023 Week 8
Search Strategy:

| **#** | **Searches** | **Results** |
| --- | --- | --- |
| 1 | virtual reality/ | 25184 |
| 2 | virtual reality exposure therapy/ | 904 |
| 3 | (virtual realit* or VR or augmented realit* or immersive technolog*).tw,kw. | 34247 |
| 4 | or/1-3 | 44191 |
| 5 | exp pain/ | 1683646 |
| 6 | exp complex regional pain syndrome/ | 11478 |
| 7 | headache/ | 275248 |
| 8 | exp "headache and facial pain"/ | 383529 |
| 9 | (pain* or neuralgia* or complex regional pain syndrome? or CRPS or headache? or head ache?).tw,kw. | 1431631 |
| 10 | or/5-9 | 2122652 |
| 11 | 4 and 10 | 2891 |
| 12 | treatment outcome/ | 946760 |
| 13 | safety/ | 270017 |
| 14 | risk assessment/ | 729878 |
| 15 | device safety/ | 16365 |
| 16 | feasibility study/ | 186240 |
| 17 | analgesia/ | 155956 |
| 18 | (effective* or feasible or feasibility or outcome? or safe or safe* or adverse event? or adverse effect? or harm* or risk? or undesirable effect? or undesirable event? or unexpected effect? or unexpected event?).tw,kw. | 10427267 |
| 19 | ((pain* or headache? or head ache?) adj3 (decreas* or manag* or reduc*)).tw,kw. | 160531 |
| 20 | or/12-19 | 11106692 |
| 21 | 4 and 10 and 20 | 2117 |
| 22 | limit 21 to english language | 2075 |
| 23 | limit 22 to dc=20220111-20231231 | 526 |
| 24 | limit 22 to dd=20220111-20231231 | 172 |
| 25 | 23 or 24 | 526 |
| 26 | remove duplicates from 25 | 514 |

**EBM Reviews - Cochrane Central Register of Controlled Trials January 2023
Search Strategy:**

| **#** | **Searches** | **Results** |
| --- | --- | --- |
| 1 | Virtual Reality Exposure Therapy/ | 265 |
| 2 | (virtual realit* or VR or augmented realit* or immersive technolog*).tw,kw. | 6249 |
| 3 | 1 or 2 | 6274 |
| 4 | exp pain/ | 61018 |
| 5 | exp Complex Regional Pain Syndromes/ | 352 |
| 6 | Headache/ | 2786 |
| 7 | exp Headache Disorders/ | 4209 |
| 8 | (pain* or neuralgia* or complex regional pain syndrome? or CRPS or headache? or head ache?).tw,kw. | 228642 |
| 9 | or/4-8 | 239874 |
| 10 | 3 and 9 | 1243 |
| 11 | Treatment Outcome/ | 164206 |
| 12 | Safety/ | 3559 |
| 13 | Risk Assessment/ | 11694 |
| 14 | Equipment Safety/ | 358 |
| 15 | feasibility studies/ | 8828 |
| 16 | Pain Management/ | 4895 |
| 17 | ((pain* or headache? or head ache?) adj3 (decreas* or manag* or reduc*)).tw,kw. | 46209 |
| 18 | or/11-17 | 219598 |
| 19 | 3 and 9 and 18 | 604 |
| 20 | limit 19 to yr="2022 - 2023" | 135 |
| 21 | remove duplicates from 20 | 135 |

**APA PsycInfo <1967 to February Week 2 2023>
Search Strategy:**

| **#** | **Searches** | **Results** |
| --- | --- | --- |
| 1 | virtual reality/ or augmented reality/ | 11207 |
| 2 | virtual reality exposure therapy/ | 246 |
| 3 | (virtual realit* or VR or augmented realit* or immersive technolog*).tw. | 11156 |
| 4 | or/1-3 | 15294 |
| 5 | exp pain/ | 64678 |
| 6 | "complex regional pain syndrome (type i)"/ | 190 |
| 7 | neuralgia/ | 548 |
| 8 | headache/ | 6865 |
| 9 | (pain* or neuralgia* or complex regional pain syndrome? or CRPS or headache? or head ache?).tw. | 133934 |
| 10 | or/5-9 | 137437 |
| 11 | 4 and 10 | 576 |
| 12 | treatment outcomes/ | 39114 |
| 13 | safety/ | 16761 |
| 14 | risk assessment/ | 15342 |
| 15 | pain management/ | 10987 |
| 16 | (effective* or feasible or feasibility or outcome? or safe or safe* or adverse event? or adverse effect? or harm* or risk? or undesirable effect? or undesirable event? or unexpected effect? or unexpected event?).tw. | 1340848 |
| 17 | ((pain* or headache? or head ache?) adj3 (decreas* or manag* or reduc*)).tw. | 20293 |
| 18 | or/12-17 | 1358081 |
| 19 | 4 and 10 and 18 | 408 |
| 20 | limit 19 to english language | 387 |
| 21 | limit 20 to yr=2022-2023 | 21 |
| 22 | remove duplicates from 21 | 21 |

**CINAHL Plus [EBSCO]**

**February 27, 2023**

| **#** | **Query** | **Limiters/Expanders** | **Last Run Via** | **Results** |
| --- | --- | --- | --- | --- |
| S19 | S3 AND S8 AND S17 | Limiters - Published Date: 20220101-20231231; English Language Search modes - Boolean/Phrase | Interface - EBSCOhost Research Databases Search Screen - Advanced Search Database - CINAHL Plus with Full Text | 132 |
| S18 | S3 AND S8 AND S17 | Search modes - Boolean/Phrase | Interface - EBSCOhost Research Databases Search Screen - Advanced Search Database - CINAHL Plus with Full Text | 566 |
| S17 | S9 OR S10 OR S11 OR S12 OR S13 OR S14 OR S15 OR S16 | Search modes - Boolean/Phrase | Interface - EBSCOhost Research Databases Search Screen - Advanced Search Database - CINAHL Plus with Full Text | 2,392,978 |
| S16 | TI ( (headache* N3 (decreas* or manag* or reduc*)) ) OR AB ( (headache* N3 (decreas* or manag* or reduc*)) ) OR TI ( ("head ache*" N3 (decreas* or manag* or reduc*)) ) OR AB ( ("head ache*" N3 (decreas* or manag* or reduc*)) ) | Search modes - Boolean/Phrase | Interface - EBSCOhost Research Databases Search Screen - Advanced Search Database - CINAHL Plus with Full Text | 2,365 |
| S15 | TI ( (pain* N3 (decreas* or manag* or reduc*)) ) OR AB ( (pain* N3 (decreas* or manag* or reduc*)) ) | Search modes - Boolean/Phrase | Interface - EBSCOhost Research Databases Search Screen - Advanced Search Database - CINAHL Plus with Full Text | 58,879 |
| S14 | TI ( (effective* or feasible or feasibility or outcome# or safe* or "adverse event#" or "adverse effect#" or harm* or risk# or "undesirable effect#" or "undesirable event#" or "unexpected effect#" or "unexpected event#") ) OR AB ( (effective* or feasible or feasibility or outcome# or safe* or "adverse event#" or "adverse effect#" or harm* or risk# or "undesirable effect#" or "undesirable event#" or "unexpected effect#" or "unexpected event#") ) | Search modes - Boolean/Phrase | Interface - EBSCOhost Research Databases Search Screen - Advanced Search Database - CINAHL Plus with Full Text | 2,144,027 |
| S13 | (MH "Pain Management") | Search modes - Boolean/Phrase | Interface - EBSCOhost Research Databases Search Screen - Advanced Search Database - CINAHL Plus with Full Text | 13,029 |
| S12 | (MH "Equipment Safety") | Search modes - Boolean/Phrase | Interface - EBSCOhost Research Databases Search Screen - Advanced Search Database - CINAHL Plus with Full Text | 4,964 |
| S11 | (MH "Risk Assessment") | Search modes - Boolean/Phrase | Interface - EBSCOhost Research Databases Search Screen - Advanced Search Database - CINAHL Plus with Full Text | 143,230 |
| S10 | (MH "Safety") | Search modes - Boolean/Phrase | Interface - EBSCOhost Research Databases Search Screen - Advanced Search Database - CINAHL Plus with Full Text | 31,374 |
| S9 | (MH "Treatment Outcomes") | Search modes - Boolean/Phrase | Interface - EBSCOhost Research Databases Search Screen - Advanced Search Database - CINAHL Plus with Full Text | 419,068 |
| S8 | S4 OR S5 OR S6 OR S7 | Search modes - Boolean/Phrase | Interface - EBSCOhost Research Databases Search Screen - Advanced Search Database - CINAHL Plus with Full Text | 428,921 |
| S7 | TI ( (pain* or neuralgia* or "complex regional pain syndrome#" or CRPS or headache# or "head ache#") ) OR AB ( (pain* or neuralgia* or "complex regional pain syndrome#" or CRPS or headache# or "head ache#") ) | Search modes - Boolean/Phrase | Interface - EBSCOhost Research Databases Search Screen - Advanced Search Database - CINAHL Plus with Full Text | 353,860 |
| S6 | (MH "Headache+") | Search modes - Boolean/Phrase | Interface - EBSCOhost Research Databases Search Screen - Advanced Search Database - CINAHL Plus with Full Text | 31,148 |
| S5 | (MH "Complex Regional Pain Syndromes+") | Search modes - Boolean/Phrase | Interface - EBSCOhost Research Databases Search Screen - Advanced Search Database - CINAHL Plus with Full Text | 2,354 |
| S4 | (MH "Pain+") | Search modes - Boolean/Phrase | Interface - EBSCOhost Research Databases Search Screen - Advanced Search Database - CINAHL Plus with Full Text | 235,393 |
| S3 | S1 OR S2 | Search modes - Boolean/Phrase | Interface - EBSCOhost Research Databases Search Screen - Advanced Search Database - CINAHL Plus with Full Text | 12,857 |
| S2 | TI ( ("virtual realit*" or VR or "augmented realit*" or "immersive technolog*") ) OR AB ( ("virtual realit*" or VR or "augmented realit*" or "immersive technolog*") ) | Search modes - Boolean/Phrase | Interface - EBSCOhost Research Databases Search Screen - Advanced Search Database - CINAHL Plus with Full Text | 8,334 |
| S1 | (MH "Virtual Reality") OR (MH "Virtual Reality Exposure Therapy") | Search modes - Boolean/Phrase | Interface - EBSCOhost Research Databases Search Screen - Advanced Search Database - CINAHL Plus with Full Text | 7,452 |
