# Supplementary material for: Extended Reality (XR) in Pediatric Acute and Chronic Pain: Systematic Review and Evidence Gap Map
Source: JMIR Pediatr Parent. 2025 Apr 7;8:e63854. doi: 10.2196/63854 (PMC12012403; doi:10.2196/63854)
Supplement: Multimedia Appendix 2 [file pediatrics_v8i1e63854_app2.docx]

**Study title:**

*Immersive Technology for acute, recurrent, or chronic pain in pediatric and adult populations: A systematic review of the safety, feasibility, and effectiveness*

**Data analysis:** The following classifications and operational definitions will be used to organize presentation of results.

**Type of pain**

1. **Acute pain - needle poke/insertion** (e.g., IV insertion, port access)
2. **Acute - wound care** (e.g., burn care, dressing changes)
3. **Acute pain – procedural** (e.g., dental procedure, minor surgery)
4. **Acute pain** **– other** (e.g., vaso occlusive crisis with sickle cell disease)
5. **Chronic pain – cancer related**
6. **Chronic pain – postsurgical/trauma**
7. **Chronic pain – headache**
8. **Chronic pain – neuropathic**
9. **Chronic pain – widespread** (e.g., Musculoskeletal, Fibromyalgia)

**Pain Targets**

1. User Experiences: Targeting
   1. **Presence** = “subjective experience of being in one place or environment, even when one is physically situated in another.”
   2. **Immersion** = can refer both to the subjective user assessment (ie, a sense of being “caught up and absorbed” in the virtual world) and to the VR system configuration (e.g., a 3-dimensional 360-degree virtual environment presented through HMD vs a 2-dimensional presentation on a computer screen).
   3. **Interactivity** = the degree to which users can influence the virtual environment, 52as facilitated by the technical configuration.
   4. **Embodiment** = “sense of having one’s body” and reflects the integration of multiple sensory signals (e.g., visual, tactile, and kinesthetic), which in turn can be manipulated by VR”
2. Cognitive
3. **Distraction** = Distraction is defined by the engagement of cognitive and attentional resources by VR stimuli, with hypoalgesic effects being driven by active competition for resources that are necessary for pain processing.
4. **CBT skills** = development of specific skills as action such as cognitive restructuring, Socratic questioning, Exposure, thought record, activity scheduling, behavioral activation, relaxation techniques, stress reduction techniques,
5. Emotional Regulation
6. **Fear/Anxiety** = fear of pain, measurement physiologically or self-report measures based on physiological state of arousal and/or diagnostic criteria for anxiety disorder (DSM-5)
7. **Depression** = intervention addressing self-report of symptoms consistent with DSM-5 diagnosis of depression
8. **Empathic Interaction** = Ability to detect the user's affective state and to respond to it in an empathic manner.
9. Social Engagement
10. Physiological
    1. **Brain Activity** = MRI, FMRI, EEG, MEG
    2. **Brain Plasticity**
    3. **Kinematic Patterns** = software/hardware promotes the patient to engage in movement patterns, physical activity and/or exercise
11. Behavioral
    1. **Pain Behavior**
12. Functioning = ability to fulfill purpose or task in a variety of relevant domains including education, social, emotional, physical, vocational,
13. Pain Intensity = observed or reported magnitude of the intensity of felt pain
14. Quality of Life = Qualitative assessment of perceived wellness across physical and mental and social domains, often assessed via self-report or parent report of health-related quality of life or generalized quality of life (e.g., pedsQL, HRQOL)
15. Healthcare Utilization
16. Safety = assessment of the adverse effects, side effects, or negative outcomes that come about as a result of use of VR
17. Feasibility ability to implement and use VR technology and level of challenge or difficulty integrating technology into intended environment or for intended purpose
    1. Satisfaction
    2. Acceptability
18. Participation / Engagement = reported or observed use of VR technology (e.g., frequency, duration, reported enjoyment)
    1. **Fun** = reported enjoyment or positive experience using the VR intervention

**Implementation Outcomes**

1. **Safety** = assessment of the adverse effects, side effects, or negative outcomes that come about as a result of use of VR
2. **Feasibility** = **Participation/Engagement** = reported or observed use of VR technology (e.g., frequency, duration, reported enjoyment)
